# The complete costs of genome sequencing: a microcosting study in cancer and rare diseases from a single center in the United Kingdom

**DOI:** 10.1038/s41436-019-0618-7

**Published:** 2019-07-30

**Authors:** Katharina Schwarze, James Buchanan, Jilles M. Fermont, Helene Dreau, Mark W. Tilley, John M. Taylor, Pavlos Antoniou, Samantha J. L. Knight, Carme Camps, Melissa M. Pentony, Erika M. Kvikstad, Steve Harris, Niko Popitsch, Alistair T. Pagnamenta, Anna Schuh, Jenny C. Taylor, Sarah Wordsworth

**Affiliations:** 1 Health Economics Research Centre, Nuffield Department of Population Health, University of Oxford, Oxford, UK; 2 National Institute for Health Research (NIHR) Oxford Biomedical Research Centre (BRC), Oxford, UK; 3 Experimental Medicine and Immunotherapeutics, Department of Medicine, University of Cambridge, Cambridge, UK; 4 Cardiovascular Epidemiology Unit, Department of Public Health & Primary Care, University of Cambridge, Cambridge, UK; 5 Molecular Diagnostics Centre, Oxford University Hospitals NHS Foundation Trust, Oxford, UK; 6 Wellcome Trust Centre for Human Genetics, University of Oxford, Oxford, UK; 7 Oxford Regional Genetics Laboratory, Oxford, UK; 8 Institute of Molecular Biotechnology (IMBA), Vienna Biocenter Campus (VBC), Vienna, Austria; 9 Department of Oncology, University of Oxford, Oxford, UK

**Keywords:** genome sequencing, cost, cancer, rare diseases, next-generation sequencing

## Abstract

**Purpose:**

The translation of genome sequencing into routine health care has been slow, partly because of concerns about affordability. The aspirational cost of sequencing a genome is $1000, but there is little evidence to support this estimate. We estimate the cost of using genome sequencing in routine clinical care in patients with cancer or rare diseases.

**Methods:**

We performed a microcosting study of Illumina-based genome sequencing in a UK National Health Service laboratory processing 399 samples/year. Cost data were collected for all steps in the sequencing pathway, including bioinformatics analysis and reporting of results. Sensitivity analysis identified key cost drivers.

**Results:**

Genome sequencing costs £6841 per cancer case (comprising matched tumor and germline samples) and £7050 per rare disease case (three samples). The consumables used during sequencing are the most expensive component of testing (68–72% of the total cost). Equipment costs are higher for rare disease cases, whereas consumable and staff costs are slightly higher for cancer cases.

**Conclusion:**

The cost of genome sequencing is underestimated if only sequencing costs are considered, and likely surpasses $1000/genome in a single laboratory. This aspirational sequencing cost will likely only be achieved if consumable costs are considerably reduced and sequencing is performed at scale.

## INTRODUCTION

Next-generation sequencing (NGS) technologies provide high-throughput simultaneous testing of multiple genes and allow either the whole genome or parts of it (via exome sequencing or targeted panels) to be sequenced in hours, at great depth and increasing sensitivity. These technologies have been in use, largely on a research basis, since 2008. Prior to 2008, the use of Sanger-based technologies meant that resequencing was substantially more expensive—for example, a human genome cost an estimated $20–25 million in 2006.^1^ With the advent of NGS, there has been a significant and ongoing decline in consumable costs, hence there is widespread expectation that a “$1000 genome” may soon be available. However, this expectation likely only reflects the consumables component and does not consider the overall costs of the sequencing process, which include sample processing (including library preparation and sequencing), bioinformatic data processing and analysis, interpretation and reporting of sequencing results, and data storage. Clinical interpretation in particular can be lengthy and costly. This dichotomy has led to descriptions of “the $1000 genome and the $100,000 analysis.”^2^

To ensure that NGS technologies are not merely an expensive addition to patient care, demand is increasing for accurate figures on the “complete” costs of the entire sequencing process. There is considerable variation in the costs reported in academic papers and the media. A review of economic evaluations of exome and genome sequencing in 2018 reported that cost estimates for a single test ranged from £382 ($555) to £3592 ($5169) for exome sequencing and from £1312 ($1906) to £17,243 ($24,810) for genome sequencing.^3^ Few cost analyses presented data transparently, and many publications did not state which components were included in cost estimates. In addition, resource use and unit costs were rarely reported in a disaggregated manner, and many studies did not calculate the actual cost of exome or genome sequencing, instead using prices charged by commercial operators. Furthermore, few studies have accounted for the number of samples that realistically must be sequenced to achieve a diagnostic result; at least two samples are required for cancer cases and three are often required for rare disease cases, with the proband and both parents often sequenced as a trio.

This paper reports a microcosting study that we undertook to provide comprehensive and detailed estimates of the complete costs of using genome sequencing to identify pathogenic variants. This study was undertaken in the context of routine care for patients with cancer or rare diseases in one National Health Service (NHS) laboratory in the United Kingdom (UK).

## MATERIALS AND METHODS

We undertook a detailed microcosting study of clinical-grade genome sequencing using the Illumina HiSeq 4000 in the Oxford Molecular Diagnostics Center (OMDC), an accredited NHS laboratory in the UK. Although clinical grade, genome sequencing is not yet routine in NHS clinical practice and the sequencing described herein was funded as part of a translational research grant. Microcosting is a highly detailed health economic costing approach in which all of the underlying resources required for an intervention or activity, such as equipment, consumables, and staff time, are identified, and then unit costs are attached to this resource use to generate an overall cost. This microcosting study was undertaken in line with the methods outlined by Drummond et al.,^4^ and was carried out from June 2016 to December 2017.

### Patient and participant recruitment

Patients with rare diseases or cancers suitable for genome sequencing were referred for sequencing via participating clinicians. Rare disease referrals were triaged at a Genomic Medicine Multi-Disciplinary Team (MDT) meeting, based on whether prior genetic testing for known genes (panel tests and arrays) had been carried out (and found to be normal). Pediatric and adult patients with a broad spectrum of rare disease (including developmental, neurological, immunological, cardiovascular, and musculoskeletal conditions) were recruited. Family trios comprising the proband and both parents were recruited where possible, since knowledge of the genetic variants and affection status of parents allows many variants to be eliminated in the filtering process. Based on our experience, this greatly improves the success rate for identifying pathogenic variants while reducing analysis time.^5^

Cancer cases were also reviewed at an MDT meeting for suitability for genome sequencing. Patients with a broad spectrum of cancer types were recruited, including breast, colorectal, prostate, and endometrial cancers. Tumor and germline DNA samples were obtained for each patient; however, tumor sequencing was only undertaken if the pathologist’s report indicated that >40% of the tissue was tumor. Following sequencing, somatic variants were identified by subtraction of the germline variants from those in the tumor.

### Genome sequencing pathway

**Fig. 1:**
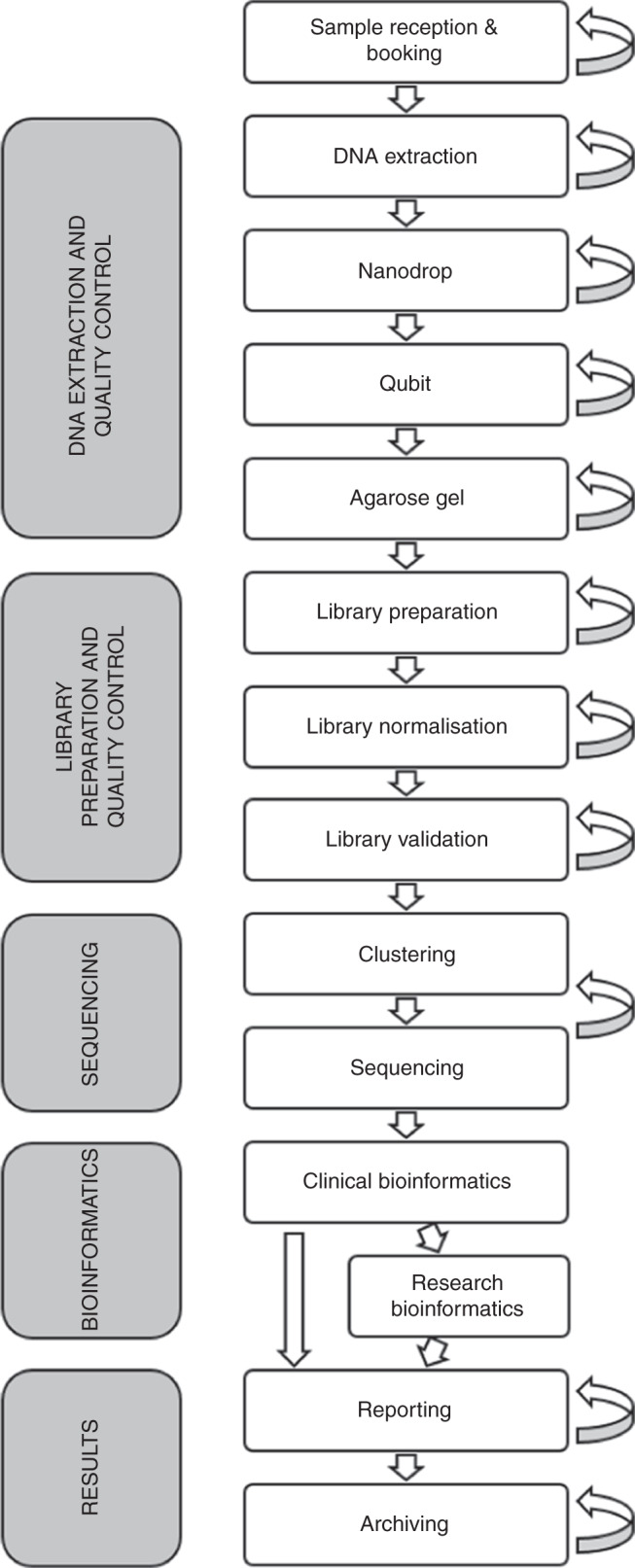
**Genome sequencing testing pathway for cancer and rare disease cases in the Oxford Molecular Diagnostics Center.** The arrows on the right-hand side indicate the potential for stage repetition due to minor process errors.

We first determined the precise testing pathway for genome sequencing in the OMDC laboratory. The standard operating procedures for the HiSeq 4000 (Illumina Inc., San Diego, CA) were used to develop costing questionnaires to collect resource use information. These questionnaires were completed by staff at the Oxford Biomedical Research Center (a public partnership between the University of Oxford and Oxford University Hospitals NHS Foundation Trust, funded by the National Institute for Health Research). All steps in the genome sequencing pathway, from sample reception to data interpretation, reporting, and archiving were considered (see Supplementary Materials—Part 1 for a detailed description of the pathway and methods). Questionnaire responses indicated that the same stages appeared in both the rare disease and cancer pathways (Fig. 1).

The bioinformatics phase included both clinical and research bioinformatics. Clinical bioinformatics analysis, which all samples passed through, consisted of a standardized pipeline to identify variants in genes known to be pathogenic for the presenting condition (see Supplementary Materials —Part 2 for details of all software packages used). For cancer cases, variants were classified as tier 1, 2, or 3 according to Li et al. and clinically actionable variants were reported.^6^ Read mapping and alignment were carried out in a similar manner for rare diseases. Annotation of rare disease variants was based on American College of Medical Genetics and Genomics (ACMG) guidelines.^7^ Variants classified as pathogenic, likely pathogenic, and of uncertain significance within the in silico panels were detailed in a clinical report. Secondary findings were also investigated in any patients or participants who had consented to this, by applying a 50-gene in silico panel as recommended by the ACMG.^8–10^

For rare disease cases where variants were not identified in known pathogenic genes for the presenting condition, we explored all genomic variants passing quality control filters and fitting the inheritance pattern and clinical features. This analysis was specific to each individual case, dependent on its complexity, duration of analysis, and requirement for functional validation studies to confirm pathogenicity. These investigations were considered within a research bioinformatics phase in this analysis, and not included in the base case costing.

At the end of the genome sequencing pathway, the results of the data analysis were reported to the referring clinician, and the sequencing data and results were archived using Arkivum (London, UK). This provided industry standard encryption, offsite storage at multiple locations, and near disk retrieval speeds.

### Resource use and unit costs

Data were collected on resource use and unit costs for each step in the pathway. This included the average staff time for each activity and salary data, use of equipment (initial costs, maintenance costs, and proportion of time used for genome sequencing diagnostics, for both computing and laboratory equipment) and consumables (laboratory supplies, software licenses, service contracts) and error rates. Resource use data were adjusted to reflect the different requirements of genome sequencing in cancer and rare diseases. In cancer cases, two DNA samples were sequenced—one extracted from the tumor tissue (TT), which required a sequencing depth of at least 75×, and one germline sample at a minimum of 30× depth. This meant that in the sequencing and bioinformatics stages, TT samples required different quantities of consumables compared with germline samples. For rare disease trios, three samples were sequenced (the proband and both parents), each at a minimum of 30× depth.

The resources that were used were then linked to their associated unit costs. Unit cost data was extracted from OMDC purchasing records where possible. If this was not possible, unit costs were obtained from commercial laboratory equipment suppliers. When cost data were only available for a kit as a whole, kit costs were apportioned equally across all items in the kit. Costs that were specified in US dollars were converted to British pounds based on the average exchange rate of $1.23:£1 in November 2016. All comparator costs reported for other studies are also presented in 2016 values (https://www.gov.uk/government/col-lections/exchange-rates-for-customs-and-vat).

The most expensive item was the HiSeq 4000 sequencing machine (Illumina), which cost £474,373, with an annual maintenance cost of £55,641. This sequencing system requires two consumable kits (a HiSeq 3000/4000 Sequencing by Synthesis [SBS] Kit costing £4207 and a HiSeq 3000/4000 Paired End [PE] Cluster Kit costing £2597), with half of each kit required per case.

Sequencing data and results were assumed to be archived in variant call format (VCF) files for five years at the end of the genome sequencing pathway. Rare disease samples were assumed to require 0.4 GB of storage space each year (i.e., 1.2 GB/year for a trio). For cancer, germline samples were assumed to require 0.4 GB per year and subtracted tumor samples were assumed to require 1 GB per year.

Information on staff salaries was extracted from national salary scales from March 2016 for NHS staff and from University of Oxford salary scales for the year 2016 for university staff (see Supplementary Materials—Part 2 for details). The midpoints of salary ranges were used, a working year was assumed to be 44 weeks, and a working week was assumed to be 37.5 hours. All salaries were inflated by 20% to incorporate National Insurance and superannuation.

### Sample throughput and error rates

The number of rare disease and cancer cases processed per year was assumed to be 224, which equated to 399 samples. This assumption was based on actual throughput at the OMDC between 1 April and 25 November 2016 (see Supplementary Materials—Part 2 for details). A small number of samples were assumed to repeat specific stages of the testing process, depending on step-specific error rates (see Supplementary Materials—Part 2 for details). These error rates have reduced over time, as experience of achieving optimal cluster density has been gained.

### Additional costing considerations

The costs of equipment items were discounted at a rate of 3.5% over a lifespan appropriate to each item. Lifespans were based on information provided by laboratory staff. If no information was available, a lifespan of five years was assumed. If maintenance costs were incurred for equipment items, these were included. Both equipment costs and maintenance costs were weighted by the percentage of time that a piece of equipment was used for genome sequencing. Sample batching was incorporated into cost calculations. As per UK guidance, value-added tax (VAT) was excluded.^11^ Total costs were inflated by an additional 20% to account for overheads, which include items such as general hospital administration, cleaning, and electricity. Further details on all resource use that was costed in this study are provided in Tables S5–S10 (Supplementary Materials—Part 3). In total, we costed 98 consumable items and 132 equipment items for cancer cases, and 86 consumable items and 125 equipment items for rare disease cases.

### Sensitivity analysis

Sensitivity analysis was performed to explore the structural and methodological uncertainty in this microcosting study and to assess how changes in key variables would affect test costs. This ensures that our results are more generalizable to other settings. Discount rates of 1.5% and 5% were evaluated, as per UK recommendations,^11^ as well as the inclusion of VAT calculated at 20%. Other parameters that were varied included the overhead rate, all inputs to staff cost calculations, the length of data archiving, kit costs, and the cost of the HiSeq 4000, whether or not rare disease cases required research bioinformatics, the size of rare disease cases, and error rates. We also evaluated the impact of archiving binary alignment map (BAM) files alongside VCF files. For both cancer and rare disease cases we assumed that this would require 150 GB of storage per year. Finally, annual throughput was varied between 100 and 2000 samples, and we tested the joint impact of both a reduction in consumable costs and an increase in sample throughput. Annual throughput of 1000 samples was tested in this analysis as this was the projected annual capacity of the HiSeq 4000 sequencing machine in this OMDC setting. The effect of increasing throughput to 2000 samples was also investigated to assess whether costs plateau when volume increases.

## RESULTS

### Cancer

Table 1 reports the costs per stage of genome sequencing for a cancer case (tumor and germline sample). The total cost of genome sequencing for a cancer case is £6841 (£3420 per genome). Consumables account for 72% of this cost (before overheads). Three-quarters (76%) of the costs of genome sequencing are accrued in the sequencing stage. Equipment costs for this stage are £615. Almost all of this cost relates to the sequencer, which costs £564 per case. Consumable costs are also high for this stage (£3688). These costs primarily relate to the use of a HiSeq SBS kit, which costs £2265 per case. The HiSeq PE kit is also a notable cost driver, costing £932 per case. The only other stage that contributes more than 10% of the total cost of genome sequencing is bioinformatics and clinical interpretation (12%). Consumables cost £267 for this stage. The main cost driver is BaseSpace Enterprise, costing £155 per case. Staff costs are higher, at £407. This is the cost associated with the use of 923 minutes (15 hours 23 minutes) of clinical scientist time per case.

**Table 1:** Cost of genome sequencing for a cancer case (tumor and germline sample)

Stage	Cost category			Total	% Total test costs before overheads
	Equipment	Consumables	Staff		
Sample reception	£0.24	£0.31	£17.59	**£18.14**	0.3%
DNA extraction	£0.19	£20.91	£15.95	**£37.06**	0.7%
Nanodrop	£0.16	£0.11	£6.20	**£6.47**	0.1%
Qubit	£0.19	£3.05	£11.20	**£14.43**	0.3%
Agarose gel	£0.15	£14.26	£22.70	**£37.11**	0.7%
Library processing^a^	£51.83	£132.09	£84.64	**£268.56**	4.7%
Sequencing^b^	£615.24	£3688.04	£48.55	**£4351.83**	76.3%
Bioinformatics	£1.80	£266.73	£406.99	**£675.52**	11.8%
Reporting	£0.00	£0.00	£257.25	**£257.25**	4.5%
Data archiving	£24.56	£0.96	£8.83	**£34.35**	0.6%
**Total (before overheads)**	**£694.35**	**£4126.46**	**£879.89**	**£5700.71**	-
% total cost	12%	72%	15%	**-**	-
**Total (including overheads calculated at 20%)**	-	-	-	**£6840.85 (£3420.43 per genome)**	-

^a^Library processing includes library preparation, normalization, and validation.

^b^Sequencing also includes clustering.

### Rare diseases

Table 2 reports the costs per stage of genome sequencing for a rare disease case (three samples). The total cost of genome sequencing such a trio is £7050 (£2350 per genome). Consumables account for 68% of this cost (before overheads). Similar to cancer cases, most of the costs of genome sequencing (79%) are accrued in the sequencing stage. Consumables cost £3688 for this stage (exactly the same cost as for cancer testing), with most of this cost again relating to the use of a HiSeq SBS kit (£2265 per case) and a HiSeq PE kit (£932 per case). Equipment costs total £923 for this stage, with almost all of this cost (£846) relating to the sequencer, as per the cancer costing. A further 7% of total costs are accrued in the bioinformatics stage. Staff time costs £313 for this stage. This is the cost associated with the use of 714 minutes (11 hours 54 minutes) of clinical scientist time per case.

**Table 2:** Cost of genome sequencing for a rare disease trio case (three samples)

Stage	Cost category			Total	% Total test costs before overheads
	Equipment	Consumables	Staff		
Sample reception	£0.35	£0.76	£21.90	**£23.01**	0.4%
DNA extraction	£0.64	£1.38	£5.72	**£7.74**	0.1%
Nanodrop	£0.24	£0.17	£7.16	**£7.56**	0.1%
Qubit	£0.28	£3.53	£17.33	**£21.14**	0.4%
Agarose gel	£0.23	£16.37	£23.27	**£39.87**	0.7%
Library processing^a^	£77.75	£197.55	£117.34	**£392.64**	6.7%
Sequencing^b^	£922.86	£3688.04	£48.55	**£4659.45**	79.3%
Bioinformatics	£2.69	£113.33	£313.22	**£429.25**	7.3%
Reporting	£0.25	£0.00	£247.69	**£247.94**	4.2%
Data archiving	£36.85	£0.82	£8.83	**£46.49**	0.8%
**Total (before overheads)**	**£1042.13**	**£4021.95**	**£811.01**	**£5875.09**	-
% total cost	18%	68%	14%	**-**	-
**Total (including overheads calculated at 20%)**	-	-	-	**£7050.11 (£2350.04 per genome)**	-

^a^Library processing includes library preparation, normalization, and validation.

^b^Sequencing also includes clustering.

### Comparing the costs for cancer and rare diseases

The use of genome sequencing as a diagnostic tool is £209 more expensive in rare diseases cases compared with cancer cases. Equipment costs are higher for rare disease cases than for cancer cases, reflecting differences in the number of samples sequenced per case and sequencing depth. Consumable and staff costs are, however, slightly higher for cancer cases. Staff costs are higher during the clinical interpretation stage for testing in cancer (£407, compared with £313 for rare disease cases) due to differences in clinical scientist time during this stage (923 minutes for cancer; 714 minutes for rare diseases), which reflects the fact that investigation of variants in previously undescribed genes is not included in the base case cost for rare disease cases (while only known cancer genes are included in the clinical analysis, their contributions to specific tumors often requires further investigation of the literature). The difference in consumable costs is driven by lower software costs for rare disease cases during the bioinformatics stage (£113, compared with £267 for cancer cases), again because consumables for investigation of variants in previously undescribed genes are not included in the base case cost for rare disease cases. Sequencing consumable costs were identical for rare disease and cancer cases because the same number of clustering and sequencing kits were used to sequence a rare disease trio (three samples at minimum 30× coverage) as a cancer case (with tumor and germline at minimum coverage of 75× and 30× respectively). Although this introduced some wastage for rare disease cases, this approach was necessary to achieve the rapid turnaround required for clinical cases. Such costs could be reduced in a high-volume facility where samples could be more effectively batched. Although equipment costs are £348 higher for rare disease cases (as 1.5× as many samples are required), this is balanced out by higher consumable and staff costs for cancer testing.

Figure S1 (Supplementary Materials—Part 4) shows the proportion of test costs accrued in each testing stage for cancer and rare disease cases. Library processing (6.7% vs. 4.7%) and sequencing (79.3% vs. 76.3%) account for a greater proportion of genome sequencing costs for rare diseases cases compared with cancer cases. Conversely, bioinformatics (11.8% vs. 7.3%) accounts for a greater proportion of genome sequencing costs for cancer cases compared with rare diseases cases.

### Sensitivity analysis

The results of the sensitivity analysis are presented in Table 3. Changes in several variables had a notable impact on the costing results. A reduction in HiSeq 4000 annual sample throughput from 399 samples to 100 samples increased the cost of cancer genome sequencing by 39% to £9500 per case, and increased the cost of rare disease genome sequencing by 53% to £10,805 per case. Increasing annual sample throughput to 1000 (the projected annual capacity of the HiSeq 4000 in this setting) reduced the cost of cancer genome sequencing and rare disease genome sequencing by 8% and 11%, respectively. Holding all other variables constant (for example, assuming no bulk discount on consumable costs), the reduction in sequencing costs that naturally occurs as throughput increases hits a floor at approximately £5950 for cancer cases (£2975 per genome) and £5796 (£1932 per genome) for rare disease cases.

**Table 3:** One-way sensitivity analysis results

Parameter (base case value)	Variation	Genome sequencing for cancer		Genome sequencing for rare diseases	
		Cost	% Change vs. base case	Cost	% Change vs. base case
**Base case analysis**	**-**	**£6840.85**	**-**	**£7050.11**	**-**
Annual sample throughput (399 samples)	100	£9499.91	39%	£10,804.71	53%
	200	£7726.24	13%	£8300.28	18%
	500	£6662.04	−3%	£6797.63	−4%
	750	£6425.56	−6%	£6463.70	−8%
	1000	£6307.31	−8%	£6296.74	−11%
	2000	£6129.95	−10%	£6046.30	−14%
National Insurance/superannuation multiplier (20%)^a^	10%	£6752.86	−1%	£6971.89	−1%
	30%	£6928.84	1%	£7128.33	1%
Weeks worked per year (44)^b^	40	£6946.44	2%	£7143.97	1%
	50	£6714.15	−2%	£6937.47	−2%
Hours worked per week (37.5)^b^	30	£7104.82	4%	£7284.77	3%
	45	£6664.87	−3%	£6893.67	−2%
Overheads (20%)	10%	£6270.78	−8%	£6462.60	−8%
	30%	£7410.92	8%	£7637.62	8%
Discount rate (3.5%)	1.5%	£6799.67	−1%	£6988.30	−1%
	5.0%	£6873.08	0%	£7098.47	1%
VAT (excluded)	Included	£7901.69	16%	£8215.43	17%
BAM files archived? (no)	Yes	£6962.87	2%	£7172.29	2%
Years of data archiving (5)	3	£6840.39	0%	£7049.71	0%
	10	£6842.00	0%	£7051.09	0%
Research bioinformatics	Excluded	£6679.23	−2%	Base^d^	-
	Included	Base^c^	-	N/A	-
	Standard case	N/A	-	£7593.67	8%
	Intermediate case	N/A	-	£7582.82	8%
PE kit cost (£2597)	50%	£6001.71	−12%	£6210.96	−12%
	150%	£7680.00	12%	£7889.25	12%
SBS kit cost (£4207)	50%	£5481.61	−20%	£5690.86	−19%
	150%	£8200.10	20%	£8409.35	19%
Sequencing machine cost (£474,373)	50%	£6669.46	−3%	£6793.01	−4%
	150%	£7012.25	3%	£7307.20	4%
Family size for rare disease cases (3)	2.4	N/A	-	£5650.39	−20%
	2.6	N/A	-	£6116.96	−13%
	2.8	N/A	-	£6583.53	−7%
Error rate—library processing (5%)	2.5%	£6834.66	0%	£7041.41	0%
	7.5%	£6847.05	0%	£7059.10	0%
Error rate—clustering (15%)	5%	£6838.92	0%	£7048.18	0%
	25%	£6842.78	0%	£7052.04	0%
Error rate for the sequencing by synthesis and paired end kits (7.7%)	4%	£6690.20	−2%	£6899.45	−2%
	12%	£7016.82	3%	£7226.08	2%
Error rate—sequencing (25%)	15%	£6835.57	0%	£7044.83	0%
	35%	£6845.95	0%	£7055.20	0%

*BAM *  binary alignment file, *PE * paired end, *SBS* sequencing by synthesis, *VAT* value-added tax.

^a^National Insurance is a UK tax that funds state benefits. As this is an expense that is directly incurred by employers (rather than a transfer payment), this cost is typically included in economic evaluations and microcosting studies.

^b^Throughput held constant in this sensitivity analysis. Hourly wage rates were generated for our analysis by combining data on annual staff salaries with assumptions regarding weeks worked per year and hours worked per week. As hourly wage rates (and thus our overall cost estimates) naturally varied when these assumptions were varied, we evaluated variations in these parameters in our sensitivity analysis.

^c^The base case analysis for cancer includes research bioinformatics costs as these costs are relatively few (£134.69). This calculation assumes that for cancer, severity of case does not impact on research bioinformatics costs.

^d^The base case analysis for rare diseases does not include research bioinformatics costs as these costs are more substantial and variable. Costs are instead provided for a standard case, requiring 120 minutes of staff time, and a case of intermediate difficulty, requiring 300 minutes of staff time.

Most changes in staff-related variables had a negligible effect on the cost of testing, as did changes in the discount rate. Including the costs of research bioinformatics for rare disease cases has a significant impact on the cost of testing. Both standard cases and cases of intermediate difficulty increase test costs by 8%, to £7594 and £7583 per case, respectively (intermediate cases require more staff time than standard cases—300 minutes per case versus 120 minutes—but require software with lower licensing costs). Varying the PE and SBS consumables kit costs by 50% above and below the base case values impacted on both test costs, but did not change the cost difference between the two testing applications. Varying the cost of the sequencer by 50% above and below the base case value changed both test costs by a negligible amount, primarily because half of the sequencer cost per cancer or rare disease case (£279 for cancer cases and £418 for rare disease cases) is the annual maintenance cost for the sequencer, not the cost of the sequencer itself. Archiving BAM files as well as VCF files increased the cost per case by just 2% for both cancer and rare disease cases.

**Fig. 2:**
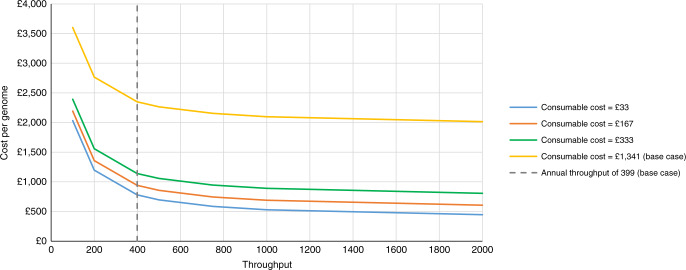
**Joint changes in annual throughput and consumable costs for genome sequencing in rare diseases (results expressed as the cost per genome).**

The full results of the joint sensitivity analysis on throughput and consumable costs are presented in Figures S2 and S3 (Supplementary Materials—Part 5) for genome sequencing in cancer and genome sequencing in rare diseases (with results expressed as the cost per case). Figure 2 presents the results of this analysis for genome sequencing in rare diseases in terms of the change in cost per genome. With an increase in annual throughput from 399 to 2000 samples, and a reduction in consumable costs from £1341 per genome to £33 per genome, the cost per genome falls from £2350 to £447.

## DISCUSSION

This paper reports the first detailed cost analysis for the complete genome sequencing process for both cancer and rare disease cases in a single center within the UK NHS. Our results show that the costs of using genome sequencing as a diagnostic tool are similar for both cancer (£6841 per case or £3420 on average per genome) and rare disease cases (£7050 per trio or £2350 per genome). Using a medium throughput sequencer (the HiSeq 4000), the key cost drivers for cancer cases are sequencing (£4352 per case, of which £3688 is for consumables) and, to a lesser extent, bioinformatics and reporting (£933). The key cost drivers for rare disease cases are the same: sequencing (£4659 per case) and bioinformatics and reporting (£677).

Although our cost estimates are similar for cancer and rare disease cases, there are some small differences in the types of costs incurred in each context based on the fact that a cancer case is considered here as two samples (tumor and germline) and a rare disease case as a trio (parents and proband). While two samples are a necessity for cancer cases to identify pathogenic variants in the tumor, a rare disease case could yield diagnostic results from the proband’s genome alone. However, based on our experience this considerably reduces the success rate for identifying pathogenic variants while also contributing substantially to analysis time and costs.^5^ Hence our policy has been to recruit rare disease trios. In this regard, it should be noted that the 100,000 Genomes Project in the UK has also elected to preferentially recruit trios for its rare disease cases. While proband-only testing is increasingly being used in some countries as an in silico panel that can be flexibly applied to multiple disorders, this restricts the potential for molecular diagnoses to those in previously identified disease genes, and makes identification of novel genes or genotype/phenotype associations unlikely.

Our cost estimates are generally lower than those reported in previous studies when considered on a per patient genome basis (the costs of also testing parents were not included in other studies).^3^ A Canadian microcosting study of genome sequencing in autism spectrum disorder cases estimated the cost of testing to be £3016 (US $4346) for a single patient sample using the Illumina HiSeq 2500 (95% confidence interval: £2866 to £3161).^12^ In this study, follow-up with Sanger sequencing for the proband and two parents was assumed to take place in 50% of cases, costing £98 (US $141) per sample (95% confidence interval: £86 to £110). A similar estimate was reported by Christensen et al. in 2018. This US study reported the results of randomized controlled trials of genome sequencing in two settings, estimating the costs of testing per patient to be £3673 (US $5338) in a cardiology setting and £3641 (US $5291) in a primary care setting.^13^ These figures included the cost of confirmatory Sanger sequencing in the proband, which ranged from £420 to £435 (US $611 to $632) per patient. Another Canadian study by Weymann et al. reported a much higher cost for the Illumina HiSeq 2500. This study estimated the costs of using genome sequencing and subsequent analysis to guide treatment decisions in advanced cancers, reporting a cost per patient of £10,820 (US $15,727; 95% confidence interval: £10,264 to £11,375) but it additionally included biopsy and tissue sample processing, RNASeq, panel testing, and experimental verification.^14^ A final Canadian microcosting study of genome sequencing in autism spectrum disorder estimated the cost of testing to be £1168 (US $1696) per genome when a trio was tested using the Illumina HiSeq X platform (95% confidence interval: £1118 to £1217).^15^ The annual cost of follow-up using Sanger sequencing, fluorescence in situ hybridization (FISH), and quantitative polymerase chain reaction (qPCR) tests in probands and their parents ranged from £46 to £51 ($66 to $75).

Our study contributes to this evidence base by presenting highly detailed cost estimates in two clinical contexts, providing full information on which components were included in these estimates. We also calculate the actual cost of genome sequencing, in contrast to previous studies that have presented a commercial price. Going forward, these cost estimates will be a useful input into economic modeling studies that underpin health technology assessments of genome sequencing, and will help to inform budgetary calculations at both the local and national level. These estimates will also be informative when determining the optimal positioning of genome sequencing in diagnostic pathways. This is a particular concern in the context of rare diseases, as these disorders are commonly characterized by diagnostic odysseys. Finally, in the UK standardized unit costs exist for many elements of health-care resource use in the form of NHS reference costs, for example.^16^ However, there are no national tariffs for genome sequencing.^17^ These cost estimates will help to fill that gap in the literature.

Several limitations of our study should, however, be noted. First, costs were calculated for the Illumina HiSeq 4000. It is therefore unclear how our estimates relate to the costs associated with alternative sequencing platforms, particularly those with higher throughput. However, as our costs are provided at a highly detailed level this should facilitate comparisons with other sequencing platforms. Second, these costs were estimated in a UK setting, so may not be directly generalizable to other country settings. However, these estimates may be broadly indicative of the magnitude of these test costs in other settings. Furthermore, the sequencing consumables unit costs are likely to be similar between countries; the discounts that are provided for bulk orders are likely to be a more significant factor in influencing cost. Studies that estimate genome sequencing costs at a similarly detailed level in other countries are urgently required, as well as studies that consider whether these cost estimates might vary in cancer or rare disease subgroups. Third, our cost estimates are based on unit costs from 2016. These unit costs will likely be different in 2019, however it was not possible to source new quotes from suppliers to update these costs. Fourth, we included the cost of overheads in our estimates by assuming that these costs were equal to 20% of the total cost of testing. This approach implies that the overheads that are attributable to sequencing are proportional to the overall cost of sequencing. Given that consumables accounted for a large proportion of sequencing costs in both cancer and rare disease cases, this assumption may not hold. Fifth, our analysis assumed a minimum sequencing depth of 75× for tumor samples, 30× for germline samples, and 30× for probands and parents sequenced as part of a rare disease trio. It was not possible to vary these assumptions in sensitivity analysis to estimate to what extent the cost of sequencing increases if sequencing depth also increases. Finally, the moratorium on the use of non-UK cloud-based storage for NHS patient data during the study period forced us to use a UK-based archiving service (Arkivum). As there are now other cloud storage options in the UK, it is possible that storage costs will be lower. However, data archiving was not a key cost driver for either application of genome sequencing.

Our results have implications for policymakers, both in the UK and in other countries. Our microcosting study was undertaken in a single laboratory processing ~400 genome sequencing samples per year, so explored the impact on cost of varying sample throughput in a single center. Lower costs are, however, likely to be achievable if genome sequencing is rolled out as a routine test in clinical practice at the national level, realizing economics of scale in terms of equipment costs and providing access to bulk discounts on consumable costs. Indeed, the “$1000 genome” may already be achievable in such a system if only sequencing consumable costs are considered. Future work evaluating the costs of sequencing at scale (particularly the link between the use of high-throughput sequencing platforms, sample throughput, and reductions in consumable costs) would help to inform decision-making at the national level regarding the appropriate configuration of sequencing services. This work is particularly relevant given the recent completion of the 100,000 Genomes Project in England and the launch of a new Genomic Medicine Service in the UK NHS.^18^ The combination of bioinformatics and reporting was the second most expensive step in the testing pathway, although these costs were much lower than those associated with the sequencing step. Further automation of the bioinformatics pipeline and streamlining of clinical reporting may reduce these costs somewhat going forward, at both the single center and national scale. However, bioinformatics and reporting costs as a proportion of overall sequencing costs may increase in the short to medium term, if automation and a reduction in sequencing consumable costs due to sequencing at scale happen at the same time. Finally, our analysis did not consider the cost of any actions taken on the basis of genome sequencing test results, such as changes in patient clinical management. Future work should explore and quantify these potentially significant downstream costs.

### Conclusions

Our findings suggest that the costs of genome sequencing and clinical analysis of a cancer case or rare disease trio are £6841 (£3420 per genome) and £7050 (£2350 per genome), respectively. The costs of sequencing are yet to meet the desired $1000 per genome figure when testing is performed on relatively small numbers of patients with cancer or a rare disease in a single center with modest throughput. Sensitivity analyses indicate that high throughput—commensurate with a national-scale facility—combined with bulk discounts on consumable costs will likely have the greatest impact on the overall cost of sequencing going forward. This will be an important consideration for policymakers in this arena.

## Supplementary information

Supplementary Materials
