# Supplementary material for: The complete costs of genome sequencing: a microcosting study in cancer and rare diseases from a single center in the United Kingdom
Source: Genet Med. 2019 Jul 30;22(1):85–94. doi: 10.1038/s41436-019-0618-7 (PMC6944636; doi:10.1038/s41436-019-0618-7)
Supplement: Supplementary file 1 — Supplementary Materials [file 41436_2019_618_MOESM1_ESM.docx]

**The complete costs of genome sequencing: A micro-costing study in cancer and rare diseases from a single centre in the United Kingdom**

**Supplementary materials**

Katharina Schwarze^1^***, James Buchanan^1,2^***, Jilles M. Fermont^3,4^, Helene Dreau^5^, Mark W. Tilley^2,6^, John C Taylor^2,7^, Pavlos Antoniou^5^, Samantha JL Knight^2,6^, Carme Camps^2,6^, Melissa M. Pentony^2,6^, Erika M. Kvikstad^2,6^, Steve Harris^2,6^, Niko Popitsch^6,8^, Alistair T. Pagnamenta^9^, Anna Schuh^5,9^, Jenny C Taylor^2,6^**** and Sarah Wordsworth^1,2^****

Corresponding author: [james.buchanan@dph.ox.ac.uk](mailto:james.buchanan@dph.ox.ac.uk)

1. Health Economics Research Centre, Nuffield Department of Population Health, University of Oxford, Oxford, UK
2. National Institute for Health Research (NIHR) Oxford Biomedical Research Centre (BRC), Oxford, UK
3. Experimental Medicine and Immunotherapeutics, Department of Medicine, University of Cambridge, Cambridge, UK
4. Cardiovascular Epidemiology Unit, Department of Public Health & Primary Care, University of Cambridge, Cambridge, UK
5. Molecular Diagnostics Centre, Oxford University Hospitals NHS Foundation Trust, Oxford, UK
6. Wellcome Trust Centre for Human Genetics, University of Oxford, Oxford, UK
7. Oxford Regional Genetics Laboratory, Oxford, UK
8. Institute of Molecular Biotechnology (IMBA), Vienna Biocenter Campus (VBC), Vienna, Austria
9. Department of Oncology, University of Oxford, Oxford, UK

**PART 1: WHOLE GENOME SEQUENCING METHODS**

**Ethical approval**

Informed consent was obtained in line with the Declaration of Helsinki and local research ethics committees. Ethical approval for cancer patients was obtained from South Central-Berkshire B Research Ethics Committee [REC no: 14/SC/1165] and for rare disease patients from the West Midlands Research Ethics Committee, as part of the MGAC Study Protocol, reference 13/WM/0466.

**DNA extraction**

On arrival, samples were booked into the laboratory computing system. Constitutional DNA was isolated from 1.5 ml peripheral blood using the QIASymphony DSP DNA Midi kit (QIAGEN Inc., USA), according to the manufacturer’s protocol. Tumour DNA was extracted from fresh frozen tissue using the All Prep Mini DNA Extraction kit (QIAGEN Inc., USA), as described in the manufacturer’s protocol. Spectrophotometric and fluorometric quantification of DNA was conducted using the Nanodrop 2000 and Qubit 2.0 Fluorometer (both ThermoFisher Scientific, Waltham, MA). Separation of DNA fragments was undertaken by agarose gel electrophoresis. DNA that passed quality control tests was then prepared for sequencing.

**Whole Genome Sequencing (WGS)**

Libraries of 350 bp fragments were generated from 1µg sheared genomic DNA using the TruSeq PCR-Free library preparation kit (Illumina Inc., San Diego, CA). 2x151 paired-end sequencing was performed using the HiSeq4000 (Illumina Inc., San Diego, CA). WGS was performed at a planned coverage of 30x for the constitutional DNA and of 75x for the tumour. The HiSeq 3000/4000 Paired End (PE) Cluster Kit and HiSeq 3000/4000 Sequencing by Synthesis (SBS) Kit were used for sequencing.

**Data Analysis: Cancer**

Paired-end alignment of sequencing data against the reference genome hg19 (GRCh37) was performed using the Whole Genome Sequencing Application v2.0, based on Isaac Alignment Tool,^1^ within BaseSpace, a cloud-based analysis tool suit (Illumina Inc., San Diego, CA).

Somatic single nucleotide (SNV) and insertion/deletion (InDel) variant calling analysis was performed using the Tumour-Normal Application v1.0, based on Strelka, within BaseSpace. Calls were annotated using VariantStudio v2 (Illumina Inc., San Diego, CA), which uses Ensembl Variant Effect Predictor (VEP) v2.8,^2^ COSMIC v77 and 1000 Genomes (v3). In a second approach, data were analysed using QIAGEN’s Ingenuity® Variant Analysis™ software (Qiagen Inc., CA). The SIFT and PolyPhen-2 algorithms were used to evaluate the impact of a mutation on protein structure or function as predicted by VEP.^3, 4^ All variants of potential clinical significance were manually inspected using integrative genomics viewer (IGV).^5^

For detection of copy number events, regions of homozygosity and copy neutral regions of homozygosity (cnLOHs), germline and paired tumour:germline Log_2_R and BAF outputs were imported, processed and calls flagged and visualised using Nexus Discovery Edition 7.5 (BioDiscovery Inc., El Segundo, California, USA).

For copy number and zygosity analyses, Log_2_R values were generated from paired and unpaired tumour and germline data and these, together with B–allele frequency (BAF) outputs, were analysed and events flagged and visualised using Nexus Discovery Edition 7.5 (BioDiscovery Inc., El Segundo, California, USA).

Translocation events were investigated using BreakDancer (v1.4.5).^6^ Analysis was limited to a set of cancer specific genes, as previously defined.^7^

**Interpretation of pathogenicity and clinical impact**

All SNVs, indels, CNAs and cnLOHs were classified with respect to their pathogenicity and clinical actionability,^8^ and information relating to germline and somatic changes at the respective locus was integrated. Analysis of germline changes in cancer genes was limited to a pre-defined *in silico* targeted panel.^9^

Several sources, including COSMIC Cancer Genes Census (v77), ‘My Cancer Genome’ (<https://www.mycancergenome.org>) and ClinicalTrials.gov (<http://clinicaltrials.gov>) were used to determine whether genetic alterations were clinically relevant. Using this information, mutations were classified as Tier 1, Tier 2 and Tier 3.^10, 11^ Tier 1 variants were defined by strong prognostic or diagnostic relevance and/or clinically actionability based on the availability of either an FDA/EMA-approved therapy or access to clinical trials relevant to the indication. Tier 2 variants were defined as those that might contribute to confirming the pathological diagnosis or have established biological relevance and/or those for which a clinical trial or approved therapy in a different tumour type was available. Tier 3 variants are those of unknown significance.

**Data Analysis: Rare Disease**

For analysis of rare disease trios, paired-end alignment and variant calling of sequencing data against the reference genome hg19 (GRCh37) was performed using the Whole Genome Sequencing Application v2.0, based on Isaac Alignment Tool,^1^ within BaseSpace, a cloud-based analysis tool suit (Illumina Inc., San Diego, CA).

A pre-defined list of known disease genes specific for the disease in question was uploaded into Illumina Variant Studio Data Analysis. Any variants in these genes were further investigated in the accredited clinical laboratory setting. All variants of potential clinical significance were manually inspected using IGV and validated by Sanger sequencing.

In the absence of variants in known genes for the disease in question, the genome sequencing reads were analysed in a research setting. Reads were re-mapped to the hs37d5 reference using Stampy.^12^ Variants were called with Platypus simultaneously in all family members,^13^ then annotated/filtered using both Ingenuity Variant Analysis and a custom analysis pipeline, VARAN, comprising VEP annotation. A frequency threshold of 1% was typically used for rare disease variants but in many cases where autosomal dominant mode of inheritance was suspected, this was reduced. Any variants of potential clinical significance were checked for frequency in gnomAD (<https://gnomad.broadinstitute.org/>) and assessed for potential deleteriousness using CADD/SIFT/PolyPhen-2. Whether a gene was intolerant to loss of function variants was assessed using the pLI metric which has recently been updated.^14, 15^ In addition to standard QC filtering, in some cases we removed variants lying in genomic loci shown to yield unreliable genotype calls across pipelines.^16^ Reporting of rare disease variants in known disease-associated genes was based on the American College of Medical Genetics guidelines.^17^ For several potentially novel candidate genes, submissions were made to GeneMatcher (<https://www.genematcher.org>) to search for similar families.^18^

**PART 2: MICRO-COSTING INPUTS**

**Table S1: Software packages used for bioinformatics**

| **Cancer** | **Rare diseases** |
| --- | --- |
| Data storage on BaseSpace | Data storage on BaseSpace |
| BaseSpace Enterprise | BaseSpace Enterprise |
| Nexus Copy Number Discovery Edition | HGMD Professional (Research bioinformatics – standard cases only) |
|  | Ingenuity Variant Analysis (Research bioinformatics – standard and intermediate cases only) |
|  | Nexus Copy Number Discovery Edition (Research bioinformatics – intermediate cases only) |

**Table S2: Unit costs applied to staff times**

| **Job title** | **Employer** | **Salary mid-point ^a^** | **Cost per hour at this mid-point** |
| --- | --- | --- | --- |
| Medical Laboratory Assistant | NHS | £21,873.00 | £13.26 |
| Medical Technical Officer | NHS | £30,222.60 | £18.32 |
| Biomedical Scientist | NHS | £31,562.40 | £19.13 |
| Clinical Scientist ^b^ / Bioinformatician | NHS | £43,653.60 | £26.46 |
| Senior Scientist | NHS | £73,477.20 | £44.53 |
| Post-doctoral Research Scientist | UOX | £54,350.40 | £32.94 |
| Consultant / Manager | UOX | £71,777.40 | £43.50 |
| Professor | UOX | £80,031.60 | £48.50 |

NHS = UK National Health Service [Source: <https://www.nhsemployers.org/~/media/Employers/Publications/Pay%20circulars/Pay-circular-AfC-1-2015.pdf>], UOX = University of Oxford [Source: <https://www1.admin.ox.ac.uk/finance/epp/payroll/scales/>]. ^a^ Includes superannuation and National Insurance. ^b^ Clinical Scientists are laboratory-based professionals responsible for performing data analysis, interpreting test results and producing reports for Senior Scientists and medical colleagues. In this study, Clinical Scientists undertook the vast majority of the bioinformatics analysis.

Note: Only the actual staff time used to complete a task was costed. No assumptions were made regarding the number of each type of staff member that would need to be employed in a laboratory in order to process the assumed sample throughput.

**Table S3: Sample throughput at the Oxford Molecular Diagnostics Centre between 1st April and 25th November 2016**

|  | **Rare diseases** | **Cancer** | **8-month total** | **12-month total** | **% of total throughput** |
| --- | --- | --- | --- | --- | --- |
| **HiSeq 2500** | 13 | 18 | 31 | 47 | 21% |
| **HiSeq 4000** | 72 | 46 | 118 | 177 | 79% |
| **Total** | 85 | 64 | 149 | 224 |  |
| **% of total throughput** | 57% | 43% |  |  |  |

**Table S4: Error rates**

| **Stage** | **Error rate (%)** |
| --- | --- |
| Sample reception | 0.9 |
| DNA extraction – rare diseases | 5.0 |
| DNA extraction – cancer germline | 10.4 |
| DNA extraction – cancer tumour | 12.0 |
| Nanodrop – spectrophotometer | 8.0 |
| Qubit – DNA quantification | 15.0 |
| Agarose gel | 0.9 |
| Library processing | 5.0 |
| Clustering | 15.0 |
| Error rate for the Sequencing by Synthesis and Paired End kits | 7.7 |
| Sequencing | 25.0 |
| Bioinformatics | 0.0 |
| Reporting and data archiving | 0.1 |

Note: The clustering and sequencing stages are connected, hence process errors do not become visible until both stages are completed. Therefore, they have the same overall error rate, as both stages must be repeated if one fails. However, within the sequencing step, the sequencing kits have a separate error rate, as these kits are replaced free-of-charge if an error arises due to a defective kit.

**PART 3: FURTHER DETAILS ON RESOURCE USE**

**Table S5: Task list and staff time by stage – genome sequencing in cancer**

| **Stage** | **Task** | **Staff job title** | **Time required per case (minutes)** |
| --- | --- | --- | --- |
| Sample reception | Sample reception | MTO | 10 |
|  | Booking in – Laboratory Information System and Specimen Reception | MTO | 10 |
|  | HICOM booking in | MTO | 5 |
|  | Booking in: NGS Laboratory Information System and Greenlight | MTO | 15 |
|  | Add on to sample tracking form | MTO | 7 |
|  | Dart and filing | MTO | 10 |
|  | Quality control of freezer temperature | MTO | 20 |
| DNA extraction - blood | Setup QIAsymphony machine | MLA | 4 |
|  | Witnessing - start | MLA | 3 |
|  | Unload elution tubes | MLA | 3 |
|  | Witnessing - stop | MLA | 3 |
|  | Daily maintenance | MLA | 3 |
|  | Monthly maintenance | MLA | 0.0 |
|  | Cleaning | MLA | 1 |
|  | Preparation | MLA | 1 |
|  | Measure | MLA | 2 |
|  | Cleaning | MLA | 1 |
| DNA extraction - tumour | Sample receipt | MTO | 5 |
|  | Prep extraction | MTO | 6 |
|  | Blend tissue | MTO | 10 |
|  | Centrifugation steps | MTO | 6 |
|  | Labelling and storage | MTO | 3 |
|  | Witnessing | MTO | 3 |
| Nanodrop | Preparation | MTO | 5 |
|  | Measure | MTO | 4 |
|  | Cleaning | MTO | 4 |
|  | Annual maintenance for Nanodrop | MTO | 2 |
|  | Cleaning | MTO | 4 |
| Qubit | Preparation | MTO | 10 |
|  | DNA sample quantification | MTO | 2 |
|  | Maintenance | MTO | 5 |
|  | Monthly maintenance | MTO | 1 |
|  | Recording results | MTO | 14 |
| Agarose gel | Sample calculation | MTO | 5 |
|  | Pipette samples into Eppendorf tubes/plate | MTO | 5 |
|  | Monthly reagent preparation | MTO | 2 |
|  | Make gel | MTO | 15 |
|  | Load gel | MTO | 10 |
|  | Run gel | MTO | 10 |
|  | Visualise gel | MTO | 10 |
|  | Clean up | MTO | 5 |
|  | Report | MTO | 10 |
|  | Weekly waste disposal | MTO | 1 |
| Library preparation | Worksheet preparation | MTO | 1 |
|  | Normalise gDNA | MTO | 4 |
|  | Second person required | MTO | 2 |
|  | Fragment DNA (Covaris) | MTO | 10 |
|  | Second person required | MTO | 1 |
|  | Clean up fragmented DNA | MTO | 17 |
|  | Second person required | MTO | 2 |
|  | Perform end repair | MTO | 7 |
|  | Remove large DNA fragments | MTO | 8 |
|  | Second person required | MTO | 1 |
|  | Remove small DNA fragments | MTO | 22 |
|  | Second person required | MTO | 2 |
|  | Add A-tailing control | MTO | 7 |
|  | Incubation | MTO | 0.4 |
|  | Add ligate | MTO | 10 |
|  | Incubation | MTO | 3 |
|  | Add stop ligation buffer | MTO | 5 |
|  | Second person required | MTO | 2 |
|  | Clean up | MTO | 31 |
| Library validation | Dilution | MTO | 8 |
|  | Reagent preparation | MTO | 19 |
|  | Running qPCR | MTO | 3 |
|  | Reporting | MTO | 13 |
|  | Sample dilution | MTO | 3 |
|  | Reagent preparation | MTO | 0.0 |
|  | Washing the bioanalyser | MTO | 1 |
|  | Pressurisation and chip preparation | MTO | 2 |
|  | Chip loading | MTO | 3 |
|  | Run bioanalyser | MTO | 2 |
|  | Maintenance | MTO | 0.2 |
|  | Reporting | MTO | 3 |
|  | Qubit preparation | MTO | 2 |
|  | Enter results into spreadsheet | MTO | 4 |
|  | Normalise and pool libraries to cluster generation | MTO | 1 |
| Library normalisation and pooling | Preparation | MTO | 8 |
|  | Normalise and pool libraries | MTO | 23 |
| Clustering | Denaturation | MTO | 5 |
|  | Master mix preparation | MTO | 5 |
|  | cBot preparation | MTO | 13 |
|  | Clustering | MTO | 15 |
|  | cBot maintenance washes | MTO | 8 |
| Sequencing | Power cycle | MTO | 5 |
|  | Water wash | MTO | 10 |
|  | Create sample sheet | MTO | 5 |
|  | Volume check and run parameters | MTO | 3 |
|  | Software preparation | MTO | 4 |
|  | Reagent preparation | MTO | 15 |
|  | Loading flow cell | MTO | 4 |
|  | Sequencing | MTO | 4 |
|  | Water wash | MTO | 10 |
|  | Maintenance wash | MTO | 5 |
|  | Record results | MTO | 8 |
|  | Quality control review | CS | 10 |
| Bioinformatics | Download .vcf files from Basespace | CS | 5 |
|  | Generation of .bed files | CS | 15 |
|  | Load the .vcf files in variant studio | CS | 2 |
|  | Annotate the variants | CS | 8 |
|  | Download BAM files | CS | 8 |
|  | BICseq tool | CS | 8 |
|  | Remove repeats | CS | 5 |
|  | Filter genes | CS | 3 |
|  | Look at affected genes | CS | 75 |
|  | Breakdancer tool | CS | 10 |
|  | Look at affected genes | CS | 15 |
|  | Gene list generation | CS | 10 |
|  | Undercover tool | CS | 3 |
|  | Report generation | CS | 30 |
|  | Analyse structural variant data | CS | 75 |
|  | Clinical confirmation - review of cancer panel data | CS | 5 |
|  | Final interpretation and report | CS | 90 |
|  | Download sequence files | CS | 8 |
|  | Alignment of sequences | CS | 15 |
|  | Run custom scripts using BAM files | CS | 3 |
|  | Apply thresholds and view in Nexus | CS | 3 |
|  | BAM (Nexus) Analysis (visualisation and curation) | CS | 60 |
|  | Filter Nexus results (acquired events) | CS | 10 |
| Reporting | Write research report | CS | 60 |
|  | Import of coverage data | CS | 10 |
|  | MDT - preparation (Clinical Scientist) | CS | 15 |
|  | MDT - meeting (Clinical Scientist) | CS | 30 |
|  | MDT - preparation (Cell Pathologist) | PDRS | 0 |
|  | MDT - meeting (Cell Pathologist) | PDRS | 30 |
|  | MDT - preparation (Manager) | Man | 30 |
|  | MDT - meeting (Manager) | Man | 30 |
|  | MDT - meeting (Bioinformatician) | Bio | 30 |
|  | MDT - meeting (Referring PI) | Cons | 30 |
|  | MDT - meeting (Consultant #1) | Cons | 30 |
|  | MDT - meeting (Consultant #2) | Cons | 30 |
|  | MDT - meeting (Consultant #3) | Cons | 30 |
|  | Amendment of report after MDT | CS | 45 |
|  | Review and authorisation of report | Prof | 30 |
|  | Distribution of report | MLA | 10 |
| Data archiving | Archive data in Arkivum | CS | 20 |

Bio = Bioinformatician; CS = Clinical scientist; Cons = Consultant; Man = Manager; MDT = multi-disciplinary team meeting; MLA = Medical laboratory assistant; MTO = Medical technical officer; PDRS = Post-doctoral research scientist; Prof = Professor; SS = Senior scientist.

**Table S6: Task list and staff time by stage – genome sequencing in rare diseases**

| **Stage** | **Task** | **Staff job title** | **Time required per case (minutes)** |
| --- | --- | --- | --- |
| Sample booking in | Sample reception | MTO | 10 |
|  | Booking in: Laboratory Information System | MTO | 10 |
|  | Transferring and storing DNA | MTO | 2 |
| Sample reception in molecular haematology | Sample reception | MTO | 10 |
|  | Booking in: Laboratory Information System | MTO | 10 |
|  | HICOM booking in | MTO | 10 |
|  | Add on to sample tracking form | MTO | 7 |
|  | Dart and filing | MTO | 10 |
|  | Transferring and storing DNA | MTO | 2 |
|  | Quality control of freezer temperature | MTO | 0.1 |
| DNA extraction | Make worklist and run setup | MLA | 3 |
|  | Blood transfers and load robot | MLA | 6 |
|  | DNA transfers | MLA | 3 |
|  | Normalisation | MLA | 6 |
|  | Witnessing | MTO | 6 |
| Nanodrop | Preparation | MTO | 5 |
|  | Measure | MTO | 6 |
|  | Cleaning | MTO | 4 |
|  | Annual maintenance for Nanodrop | MTO | 3 |
|  | Cleaning | MTO | 4 |
| Qubit | Preparation | MTO | 10 |
|  | DNA sample quantification | MTO | 3 |
|  | Maintenance | MTO | 5 |
|  | Monthly maintenance | MTO | 1 |
|  | Recording results | MTO | 30 |
| Agarose gel | Sample calculation | MTO | 5 |
|  | Pipette samples into Eppendorf tubes/plate | MTO | 5 |
|  | Monthly reagent preparation | MTO | 4 |
|  | Make gel | MTO | 15 |
|  | Load gel | MTO | 10 |
|  | Run gel | MTO | 10 |
|  | Visualise gel | MTO | 10 |
|  | Clean up | MTO | 5 |
|  | Report | MTO | 10 |
|  | Weekly waste disposal | MTO | 2 |
| Library preparation | Worksheet preparation | MTO | 2 |
|  | Normalise gDNA | MTO | 7 |
|  | Second person required | MTO | 3 |
|  | Fragment DNA (Covaris) | MTO | 15 |
|  | Second person required | MTO | 2 |
|  | Clean up fragmented DNA | MTO | 25 |
|  | Second person required | MTO | 3 |
|  | Perform end repair | MTO | 10 |
|  | Remove large DNA fragments | MTO | 12 |
|  | Second person required | MTO | 2 |
|  | Remove small DNA fragments | MTO | 33 |
|  | Second person required | MTO | 3 |
|  | Add A-tailing control | MTO | 11 |
|  | Incubation | MTO | 1 |
|  | Add ligate | MTO | 15 |
|  | Incubation | MTO | 4 |
|  | Add stop ligation buffer | MTO | 8 |
|  | Second person required | MTO | 3 |
|  | Clean up | MTO | 47 |
| Library validation | Dilution | MTO | 12 |
|  | Reagent preparation | MTO | 28 |
|  | Running qPCR | MTO | 5 |
|  | Reporting | MTO | 20 |
|  | Sample dilution | MTO | 5 |
|  | Reagent preparation | MTO | 0.1 |
|  | Washing the bioanalyser | MTO | 2 |
|  | Pressurisation and chip preparation | MTO | 3 |
|  | Chip loading | MTO | 5 |
|  | Run bioanalyser | MTO | 3 |
|  | Maintenance | MTO | 0.3 |
|  | Reporting | MTO | 5 |
|  | Qubit preparation | MTO | 3 |
|  | Enter results into spreadsheet | MTO | 7 |
|  | Normalise and pool libraries to cluster generation | MTO | 2 |
| Library normalisation and pooling | Preparation | MTO | 15 |
|  | Normalise and pool libraries | MTO | 45 |
| Clustering | Denaturation | MTO | 5 |
|  | Master mix preparation | MTO | 5 |
|  | cBot preparation | MTO | 13 |
|  | Clustering | MTO | 15 |
|  | cBot maintenance washes | MTO | 8 |
| Sequencing | Power cycle | MTO | 5 |
|  | Water wash | MTO | 10 |
|  | Create sample sheet | MTO | 5 |
|  | Volume check and run parameters | MTO | 3 |
|  | Software preparation | MTO | 4 |
|  | Reagent preparation | MTO | 15 |
|  | Loading flow cell | MTO | 4 |
|  | Sequencing | MTO | 4 |
|  | Water wash | MTO | 10 |
|  | Maintenance wash | MTO | 5 |
|  | Record results | MTO | 8 |
|  | Quality control review | CS | 10 |
| Bioinformatics | Download .vcf files from Basespace | CS | 10 |
|  | Generation of .bed files | CS | 45 |
|  | Load the .vcf files in variant studio | CS | 9 |
|  | Annotate the variants | CS | 15 |
|  | Download BAM files | CS | 15 |
|  | BICseq tool | CS | 15 |
|  | Remove repeats | CS | 10 |
|  | Filter genes | CS | 5 |
|  | Look at affected genes | CS | 150 |
|  | Breakdancer tool | CS | 20 |
|  | Look at affected genes | CS | 30 |
|  | Gene list generation | CS | 20 |
|  | Undercover tool | CS | 5 |
|  | Report generation | CS | 60 |
|  | Analyse structural variant data | CS | 60 |
|  | Investigate variants | CS | 120 |
|  | Clinical confirmation | CS | 60 |
|  | Activate samples | CS | 15 |
|  | Further analysis (e.g. MLPA) | CS | 30 |
|  | Second check of variants confirmation analysis | CS | 20 |
| Reporting | Write research report | CS | 30 |
|  | Import of coverage data | CS | 10 |
|  | Clinical confirmation report | CS | 90 |
|  | Additional findings report | CS | 90 |
|  | Review and authorisation of report | PDRS | 30 |
|  | Distribution of report | MLA | 10 |
| Data archiving | Archive data in Arkivum | CS | 20 |

Bio = Bioinformatician; CS = Clinical scientist; Cons = Consultant; Man = Manager; MDT = multi-disciplinary team meeting; MLA = Medical laboratory assistant; MTO = Medical technical officer; PDRS = Post-doctoral research scientist; Prof = Professor; SS = Senior scientist.

**Table S7: Consumables used by stage – genome sequencing in cancer**

| **Stage** | **Type of consumable** | **Units used per case** |
| --- | --- | --- |
| Sample reception | Laboratory Information System labels | 2 |
|  | Brady spot labels | 2 |
| DNA extraction - blood | QIAsymphony DSP midi kit | 0.1 |
|  | QIAsymphony tips (200uL) | 0.02 |
|  | QIAsymphony tips (1500uL) | 0.6 |
|  | Cartridge wells | 0.1 |
|  | Tip waste bags | 0.001 |
|  | Rod covers | 0.02 |
|  | Sigma ultra pure water (200uL) | 0.03 |
|  | Elution tube (2 mL Screw cap) | 0.1 |
|  | Screw cap | 0.1 |
|  | Qiagen EB buffer (μl) | 1.3 |
|  | P10 pipette tips | 2 |
|  | Sigma molecular grade water (ml) | 2.3 |
| DNA extraction - tumour | Tissue rupture probe | 1 |
|  | P200 pipette tips | 2 |
|  | P1000 pipette tips | 5 |
|  | 1.5ml Eppendorf tubes | 2 |
|  | 2-Mercaptoethanol | 0.03 |
|  | Allprep mini-kit | 1 |
|  | Buffer RLT+ | 1.5 |
| Nanodrop | Qiagen EB buffer (μl) | 1.3 |
|  | P10 pipette tips | 2 |
|  | Sigma molecular grade water (ml) | 2.3 |
| Qubit | 0.5ml Eppendorf tubes | 6 |
|  | 1.5ml Eppendorf tubes | 0.7 |
|  | Qubit broad-range DNA kit | 7 |
|  | P10 pipette tips | 6 |
|  | P20 pipette tips | 4 |
|  | P200 pipette tips | 1.3 |
|  | P1000 pipette tips | 0.7 |
| Agarose gel | Ethyl bromide (ul) | 3 |
|  | 0.5ml Eppendorf tubes | 2 |
|  | 1.5ml Eppendorf tubes | 0.7 |
|  | P10 pipette tips | 4.7 |
|  | P20 pipettes tips | 4 |
|  | TB buffer (ml) | 11.7 |
|  | Ladder HiPair 1 (ml) | 3.3 |
|  | Gel Dye - Ficoll | 2 |
|  | Thermal printer paper | 2 |
|  | Gel Dye - Bromophenol | 0.2 |
|  | Gel Dye - Xylene Cyanole | 0.4 |
|  | NuSieve agarose | 3 |
| Library preparation | Truseq DNA PCR-Free sample preparation Low Throughput kit | 0.7 |
|  | 25ml glass pipette | 0.4 |
|  | P10 pipette tips | 24.4 |
|  | P20 pipette tips | 15.6 |
|  | P200 pipette tips | 100 |
|  | P1000 pipette tips | 0.4 |
|  | Covaris tubes | 2 |
|  | EtOH (ml) | 2.9 |
|  | 1.5 Eppendorp tubes | 46.7 |
|  | Microseal 'B' adhesive seal | 3.3 |
|  | PCR plates | 0.2 |
|  | Sigma molecular grade water (ml) | 2.7 |
| Library validation | 0.5ml Eppendorf tubes | 5 |
|  | 1.5ml Eppendorf tubes | 6.7 |
|  | 2ml tubes | 2.2 |
|  | P10 pipette tips | 40 |
|  | P20 pipette tips | 13.5 |
|  | P200 pipette tips | 0.9 |
|  | P1000 pipette tips | 2.2 |
|  | Optical seals | 0.2 |
|  | ABI plates | 0.2 |
|  | Kapa library quantification kit | 0.04 |
|  | Agilent high sensitivity DNA Kit | 0.2 |
|  | Water sigma (ml) | 0.2 |
|  | RNA free water (μl) | 177.8 |
| Library normalisation and pooling | P10 pipette tips | 3.5 |
|  | P20 pipette tips | 8 |
|  | 1.5ml Eppendorf tubes | 10 |
|  | Phix control kit (μl) | 1 |
| Clustering | P10 pipette tips | 9 |
|  | P20 pipette tips | 2 |
|  | P200 pipette tips | 8 |
|  | P1000 pipette tips | 3 |
|  | 1.5ml Eppendorf tubes | 3 |
|  | Optical tissues | 1.25 |
|  | NaOH 10M | 0.01 |
|  | UltraPure 1M Tris-HCl, pH 8.0 | 0.1 |
|  | Strip tubes | 0.5 |
|  | Flat PCR caps | 1 |
|  | HiSeq 4000 Kit (Paired ends) / Box 1 & FC (Cluster kit) | 1 |
| Sequencing | 15ml Falcons | 10.5 |
|  | 50ml Falcons | 0.5 |
|  | 250ml Corning bottles | 8.5 |
|  | Proclin (ml) | 0.1 |
|  | Tween 20 (ml) | 1.0 |
|  | HiSeq 4000 Kit (Paired ends) / Cluster kit | 0.5 |
|  | HiSeq 4000 Kit (Sequencing by Synthesis) | 0.5 |
|  | Optical tissues | 2.5 |
| Bioinformatics | Data storage on Basespace (GL - 30X) | 59.6 |
|  | Data storage on Basespace (Tumour - 75X) | 157.2 |
|  | Basespace Enterprise (Isaac - GL) | 25 |
|  | Basespace Enterprise (Isaac - Tumour) | 72 |
|  | Basespace Enterprise (Strelka computing) | 93 |
|  | Basespace Enterprise (Membership of cloud domain) | 2 |
| Data archiving | Arkivum | - |

**Table S8: Consumables used by stage – genome sequencing in rare diseases**

| **Stage** | **Type of consumable** | **Units used per case** |
| --- | --- | --- |
| Sample booking in | Laboratory Information System labels | 3 |
| Sample reception in molecular haematology | Laboratory Information System labels | 3 |
|  | Brady spot labels | 3 |
| DNA extraction | Ethanol 70% | 0.9 |
|  | Cell lysis sol | 0.9 |
|  | DNA hydration sol | 0.1 |
|  | Precipitation | 0.3 |
|  | Qubes | 0.4 |
|  | Red blood cell lysis | 3 |
|  | Isopropanol | 1.9 |
|  | FluidX storage tube | 0.2 |
|  | Pasteur pipette | 6 |
| Nanodrop | Qiagen EB buffer (μl) | 2 |
|  | P10 pipette tips | 3 |
|  | Sigma molecular grade water (ml) | 3.5 |
| Qubit | 0.5ml Eppendorf tubes | 6 |
|  | 1.5ml Eppendorf tubes | 1 |
|  | Qubit broad-range DNA kit | 8 |
|  | P10 pipette tips | 7 |
|  | P20 pipette tips | 5 |
|  | P200 pipette tips | 2 |
|  | P1000 pipette tips | 1 |
| Agarose gel | Ethyl bromide (ul) | 3 |
|  | 0.5ml Eppendorf tubes | 3 |
|  | 1.5ml Eppendorf tubes | 1 |
|  | P10 pipette tips | 5 |
|  | P20 pipettes tips | 5 |
|  | TB buffer (ml) | 11.7 |
|  | Ladder HiPair 1 (ml) | 3.3 |
|  | Gel Dye - Ficoll | 3 |
|  | Thermal printer paper | 3 |
|  | Gel Dye - Bromophenol | 0.3 |
|  | Gel Dye - Xylene Cyanole | 0.6 |
|  | NuSieve agarose | 3 |
| Library preparation | Truseq DNA PCR-Free sample preparation Low Throughput kit | 1 |
|  | 25ml glass pipette | 0.7 |
|  | P10 pipette tips | 36.7 |
|  | P20 pipette tips | 23.3 |
|  | P200 pipette tips | 150 |
|  | P1000 pipette tips | 0.7 |
|  | Covaris tubes | 3 |
|  | EtOH (ml) | 4.3 |
|  | 1.5 Eppendorp tubes | 70 |
|  | Microseal 'B' adhesive seal | 5 |
|  | PCR plates | 0.3 |
|  | Sigma molecular grade water (ml) | 4 |
| Library validation | 0.5ml Eppendorf tubes | 6 |
|  | 1.5ml Eppendorf tubes | 7 |
|  | 2ml tubes | 3.3 |
|  | P10 pipette tips | 60 |
|  | P20 pipette tips | 20.3 |
|  | P200 pipette tips | 1.3 |
|  | P1000 pipette tips | 2.7 |
|  | Optical seals | 0.3 |
|  | ABI plates | 0.3 |
|  | Kapa library quantification kit | 0.1 |
|  | Agilent high sensitivity DNA Kit | 0.3 |
|  | Water sigma (ml) | 0.3 |
|  | RNA free water (μl) | 266.7 |
| Library normalisation and pooling | P10 pipette tips | 3.5 |
|  | P20 pipette tips | 8 |
|  | 1.5ml Eppendorf tubes | 10 |
|  | Phix control kit (μl) | 1 |
| Clustering | P10 pipette tips | 9 |
|  | P20 pipette tips | 2 |
|  | P200 pipette tips | 8 |
|  | P1000 pipette tips | 3 |
|  | 1.5ml Eppendorf tubes | 3 |
|  | Optical tissues | 1.25 |
|  | NaOH 10M | 0.005 |
|  | UltraPure 1M Tris-HCl, pH 8.0 | 0.1 |
|  | Strip tubes | 0.5 |
|  | Flat PCR caps | 1 |
|  | HiSeq 4000 Kit (Paired ends) / Box 1 & FC (Cluster kit) | 1 |
| Sequencing | 15ml Falcons | 10.5 |
|  | 50ml Falcons | 0.5 |
|  | 250ml Corning bottles | 8.5 |
|  | Proclin (ml) | 0.1 |
|  | Tween 20 (ml) | 1.0 |
|  | HiSeq 4000 Kit (Paired ends) / Cluster kit | 0.5 |
|  | HiSeq 4000 Kit (Sequencing by Synthesis) | 0.5 |
|  | Optical tissues | 2.5 |
| Bioinformatics | Data storage on Basespace | 178.7 |
|  | Basespace Enterprise (Isaac - RD) | 75 |
|  | Basespace Enterprise (Membership of cloud domain) | 3 |
| Data archiving | Arkivum | - |

**Table S9: Equipment used by stage – genome sequencing in cancer**

| **Stage** | **Type of equipment** | **Quantity used** | **Percentage of equipment time used for genome sequencing** |
| --- | --- | --- | --- |
| Sample reception | Computer | 2 | 1% and 5% |
|  | Printer | 1 | 1% |
|  | Reception buckets | 1 | 100% |
|  | Dart scanner | 1 | 1% |
|  | Box system | 4 | 100% |
|  | Fridge | 3 | 13% |
|  | Freezer | 6 | 1% |
|  | Freezer trays | 1 | 100% |
|  | Thermometer (room temperature) | 4 | 13% |
|  | Thermometer (cold storage) | 9 | 5% |
|  | Scanner | 1 | 1% |
|  | Laptop | 1 | 1% |
| DNA extraction - blood | QIA Symphony Machine (including racks) | 1 | 1% |
|  | Vortexer | 1 | 3% |
|  | P10 pipette | 1 | 5% |
|  | Computer | 1 | 3% |
|  | Nanodrop 2000 | 1 | 3% |
|  | Centrifuge | 1 | 1% |
| DNA extraction - tumour | Laboratory blender | 1 | 1% |
|  | Centrifuge | 1 | >1% |
|  | Vortexer | 1 | 3% |
|  | Centrifuge | 1 | 1% |
|  | P200 pipette | 1 | 5% |
|  | P1000 pipette | 1 | 5% |
|  | Eppendorf racks | 1 | 5% |
| Nanodrop | P10 pipette | 1 | 5% |
|  | Computer | 1 | 3% |
|  | Printer | 1 | 1% |
|  | Nanodrop 2000 | 1 | 3% |
|  | Vortexer | 1 | 3% |
|  | Centrifuge | 1 | 1% |
| Qubit | Qubit Fluorometer | 1 | 5% |
|  | Vortexer | 1 | 3% |
|  | Microcentrifuge | 1 | 1% |
|  | P2 pipette | 1 | 5% |
|  | P10 pipette | 1 | 5% |
|  | P20 pipette | 1 | 5% |
|  | P200 pipette | 1 | 5% |
|  | P1000 pipette | 1 | 5% |
|  | Centrifuge | 1 | 3% |
|  | Eppendorf racks | 1 | 5% |
|  | Fridge | 1 | 3% |
|  | Computer | 1 | 5% |
| Agarose gel | Microwave | 1 | 1% |
|  | Gel tank | 1 | 5% |
|  | Electrophoresis leads pair | 1 | 5% |
|  | Electrophoresis powerpack | 1 | 5% |
|  | P10 pipette | 1 | 15% |
|  | P20 pipette | 1 | 1% |
|  | BioRad Gel Doc XR | 1 | 1% |
|  | Carboy tank | 1 | 5% |
|  | Water purifier | 1 | 1% |
|  | Flasks | 1 | 100% |
|  | Printer | 1 | 1% |
|  | Computer | 1 | 2% |
|  | Printer | 1 | 1% |
|  | Centrifuge | 1 | 1% |
|  | Gel combs | 1 | 5% |
|  | Gel cast | 1 | 5% |
| Library preparation | Computer | 2 | 1% and 2% |
|  | Printer | 2 | 1% |
|  | P10 pipette | 1 | 20% |
|  | P20 pipette | 1 | 10% |
|  | P200 pipette | 1 | 50% |
|  | P1000 pipette | 1 | 60% |
|  | Electronic pipette | 1 | 40% |
|  | Centrifuge | 2 | 5% and 50% |
|  | Microcentrifuge | 1 | 10% |
|  | Freezer | 2 | 1% and 25% |
|  | Covaris ultrasonicator | 1 | 25% |
|  | Covaris adapter | 1 | 100% |
|  | Thermal cycler | 1 | 80% |
|  | Vortexer Nanodrop/Qubit | 1 | 1% |
|  | Vortexer | 1 | 25% |
|  | Tube rack | 1 | 10% |
|  | Magnetic stand | 1 | 80% |
|  | Archiving box | 3 | 100% |
|  | Axygen microtube racks | 4 | 100% |
|  | Carboy | 3 | 100% |
| Library validation | Bioanalyser | 1 | 80% |
|  | Computer | 2 | 10% and 80% |
|  | Printer | 1 | 1% |
|  | P2 pipette | 2 | 5% and 10% |
|  | P10 pipette | 2 | 5% and 10% |
|  | P20 pipiette | 2 | 1% and 10% |
|  | P200 pipette | 1 | 10% |
|  | P1000 pipette | 2 | 5% and 10% |
|  | Laptop | 1 | 50% |
|  | qPCR analyser | 1 | 50% |
|  | Eppendorf racks | 2 | 5% |
|  | Fridge | 1 | 3% |
|  | Plate centrifuge | 1 | 1% |
|  | Vortexer (Bioanalyser) | 1 | 80% |
|  | Vortexer Nanodrop/Qubit | 1 | 1% |
|  | Vortexer | 1 | 3% |
|  | Pressuriser syringe holder | 1 | 80% |
|  | Microcentrifuge | 1 | 10% |
| Library normalisation and pooling | P10 pipette | 1 | 30% |
|  | P20 pipette | 1 | 30% |
|  | Computer | 1 | 1% |
|  | Printer | 1 | 1% |
|  | Vortexer | 1 | 30% |
|  | Centrifuge | 1 | 30% |
|  | Rack | 1 | 30% |
|  | Icebucket | 1 | 30% |
|  | Freezer (small) | 1 | 30% |
| Clustering | P10 pipette | 1 | 30% |
|  | P20 pipette | 1 | 30% |
|  | P200 pipette | 1 | 30% |
|  | P1000 pipette | 1 | 30% |
|  | Vortexer | 1 | 30% |
|  | Centrifuge | 1 | 30% |
|  | Rack | 1 | 30% |
|  | Icebucket | 1 | 30% |
|  | Freezer (small) | 1 | 30% |
|  | cBOT | 1 | 100% |
| Sequencing | HiSeq 4000 | 1 | 100% |
|  | Fridge (flow cell) | 1 | 50% |
|  | Fridge (defrosting) | 1 | 50% |
|  | Metal trays | 1 | 100% |
|  | 15ml racks | 1 | 100% |
|  | Measuring flask | 1 | 100% |
|  | Computer | 1 | 50% |
|  | Printer | 1 | 100% |
|  | Carboy tank | 2 | 50% and 100% |
|  | Tweezers | 1 | 100% |
| Bioinformatics | Monitor | 3 | 50% |
|  | Computer | 3 | 50% |
| Reporting | Computer | 1 | 15% |
| Data archiving | Arkivum installation | 1 | 100% |
|  | Arkivum license fee | 1 | 100% |
|  | Computer | 1 | 15% |

**Table S10: Equipment used by stage – genome sequencing in rare diseases**

| **Stage** | **Type of equipment** | **Quantity used** | **Percentage of equipment time used for genome sequencing** |
| --- | --- | --- | --- |
| Sample booking in | Computer | 1 | >1% |
|  | Printer | 1 | >1% |
|  | Reception buckets | 1 | >1% |
|  | Fridge | 1 | >1% |
|  | Thermometer (room temperature) | 4 | >1% |
|  | Thermometer (cold storage) | 9 | >1% |
| Sample reception in molecular haematology | Computer | 2 | 1% and 5% |
|  | Printer | 1 | 1% |
|  | Reception buckets | 1 | 100% |
|  | Dart scanner | 1 | 1% |
|  | Box system | 4 | 100% |
|  | Fridge | 3 | 13% |
|  | Freezer | 6 | 1% |
|  | Freezer trays | 1 | 100% |
|  | Thermometer (room temperature) | 4 | 13% |
|  | Thermometer (cold storage) | 9 | 5% |
| DNA extraction | Computer | 1 | 5% |
|  | Qiagen autopure LS robot | 1 | <1% |
| Nanodrop | P10 pipette | 1 | 5% |
|  | Computer | 1 | 3% |
|  | Printer | 1 | 1% |
|  | Nanodrop 2000 | 1 | 3% |
|  | Vortexer | 1 | 3% |
|  | Centrifuge | 1 | 1% |
| Qubit | Qubit Fluorometer | 1 | 5% |
|  | Vortexer | 1 | 3% |
|  | Microcentrifuge | 1 | 1% |
|  | P2 pipette | 1 | 5% |
|  | P10 pipette | 1 | 5% |
|  | P20 pipette | 1 | 5% |
|  | P200 pipette | 1 | 5% |
|  | P1000 pipette | 1 | 5% |
|  | Centrifuge | 1 | 3% |
|  | Eppendorf racks | 1 | 5% |
|  | Fridge | 1 | 3% |
|  | Computer | 1 | 5% |
| Agarose gel | Microwave | 1 | 1% |
|  | Gel tank | 1 | 5% |
|  | Electrophoresis leads pair | 1 | 5% |
|  | Electrophoresis powerpack | 1 | 5% |
|  | P10 pipette | 1 | 15% |
|  | P20 pipette | 1 | 1% |
|  | BioRad Gel Doc XR | 1 | 1% |
|  | Carboy tank | 1 | 5% |
|  | Water purifier | 1 | 1% |
|  | Flasks | 1 | 100% |
|  | Printer | 1 | 1% |
|  | Computer | 1 | 2% |
|  | Printer | 1 | 1% |
|  | Centrifuge | 1 | 1% |
|  | Gel combs | 1 | 5% |
|  | Gel cast | 1 | 5% |
| Library preparation | Computer | 2 | 1% and 2% |
|  | Printer | 2 | 1% |
|  | P10 pipette | 1 | 20% |
|  | P20 pipette | 1 | 10% |
|  | P200 pipette | 1 | 50% |
|  | P1000 pipette | 1 | 60% |
|  | Electronic pipette | 1 | 40% |
|  | Centrifuge | 2 | 5% and 50% |
|  | Microcentrifuge | 1 | 10% |
|  | Freezer | 2 | 1% and 25% |
|  | Covaris ultrasonicator | 1 | 25% |
|  | Covaris adapter | 1 | 100% |
|  | Thermal cycler | 1 | 80% |
|  | Vortexer Nanodrop/Qubit | 1 | 1% |
|  | Vortexer | 1 | 25% |
|  | Tube rack | 1 | 10% |
|  | Magnetic stand | 1 | 80% |
|  | Archiving box | 3 | 100% |
|  | Axygen microtube racks | 4 | 100% |
|  | Carboy | 3 | 100% |
| Library validation | Bioanalyser | 1 | 80% |
|  | Computer | 2 | 10% and 80% |
|  | Printer | 1 | 1% |
|  | P2 pipette | 2 | 5% and 10% |
|  | P10 pipette | 2 | 5% and 10% |
|  | P20 pipiette | 2 | 1% and 10% |
|  | P200 pipette | 1 | 10% |
|  | P1000 pipette | 2 | 5% and 10% |
|  | Laptop | 1 | 50% |
|  | qPCR analyser | 1 | 50% |
|  | Eppendorf racks | 2 | 5% |
|  | Fridge | 1 | 3% |
|  | Plate centrifuge | 1 | 1% |
|  | Vortexer (Bioanalyser) | 1 | 80% |
|  | Vortexer Nanodrop/Qubit | 1 | 1% |
|  | Vortexer | 1 | 3% |
|  | Pressuriser syringe holder | 1 | 80% |
|  | Microcentrifuge | 1 | 10% |
| Library normalisation and pooling | P10 pipette | 1 | 30% |
|  | P20 pipette | 1 | 30% |
|  | Computer | 1 | 1% |
|  | Printer | 1 | 1% |
|  | Vortexer | 1 | 30% |
|  | Centrifuge | 1 | 30% |
|  | Rack | 1 | 30% |
|  | Icebucket | 1 | 30% |
|  | Freezer (small) | 1 | 30% |
| Clustering | P10 pipette | 1 | 30% |
|  | P20 pipette | 1 | 30% |
|  | P200 pipette | 1 | 30% |
|  | P1000 pipette | 1 | 30% |
|  | Vortexer | 1 | 30% |
|  | Centrifuge | 1 | 30% |
|  | Rack | 1 | 30% |
|  | Icebucket | 1 | 30% |
|  | Freezer (small) | 1 | 30% |
|  | cBOT | 1 | 100% |
| Sequencing | HiSeq 4000 | 1 | 100% |
|  | Fridge (flow cell) | 1 | 50% |
|  | Fridge (defrosting) | 1 | 50% |
|  | Metal trays | 1 | 100% |
|  | 15ml racks | 1 | 100% |
|  | Measuring flask | 1 | 100% |
|  | Computer | 1 | 50% |
|  | Printer | 1 | 100% |
|  | Carboy tank | 2 | 50% and 100% |
|  | Tweezers | 1 | 100% |
| Bioinformatics | Monitor | 3 | 50% |
|  | Computer | 3 | 50% |
| Reporting | Computer | 1 | 15% |
| Data archiving | Arkivum installation | 1 | 100% |
|  | Arkivum license fee | 1 | 100% |
|  | Computer | 1 | 15% |

**PART 4: RESULTS – COST DIFFERENCES BETWEEN CANCER AND RARE DISEASES**

**Figure S1: Proportion of test costs accrued in each testing stage for the two applications**

Note: Library processing includes preparation, normalisation and validation. Sequencing includes both sequencing and clustering.

**PART 5: SENSITIVITY ANALYSIS RESULTS**

**Figure S2: Joint changes in annual throughput and consumable costs for genome sequencing in cancer (results expressed as the cost per case)**

**Figure S3: Joint changes in annual throughput and consumable costs for genome sequencing in rare diseases (results expressed as the cost per case)**

**REFERENCES**

1. Raczy C, Petrovski R, Saunders CT et al. Isaac: ultra-fast whole-genome secondary analysis on Illumina sequencing platforms. *Bioinformatics* 2013; **29**: 2041-3.

2. McLaren W, Gil L, Hunt SE et al. The Ensembl Variant Effect Predictor. *Genome Biology* 2016; **17**: 122.

3. Kumar P, Henikoff S, Ng PC. Predicting the effects of coding non-synonymous variants on protein function using the SIFT algorithm. *Nature Protocols* 2009; **4**: 1073.

4. Adzhubei IA, Schmidt S, Peshkin L et al. A method and server for predicting damaging missense mutations. *Nature methods* 2010; **7**: 248-9.

5. Robinson JT, Thorvaldsdóttir H, Winckler W et al. Integrative genomics viewer. *Nature biotechnology* 2011; **29**: 24-6.

6. Chen K, Wallis JW, McLellan MD et al. BreakDancer: an algorithm for high-resolution mapping of genomic structural variation. *Nature Methods* 2009; **6**: 677.

7. Huret J-L, Ahmad M, Arsaban M et al. Atlas of genetics and cytogenetics in oncology and haematology in 2013. *Nucleic acids research* 2013; **41**: D920-D4.

8. Futreal PA, Coin L, Marshall M et al. A census of human cancer genes. *Nature reviews Cancer* 2004; **4**: 177-83.

9. Schuh A, Dreau H, Knight SJL et al. Clinically actionable mutation profiles in patients with cancer identified by whole-genome sequencing. *Cold Spring Harbor molecular case studies*; **4**: a002279.

10. Sukhai MA, Craddock KJ, Thomas M et al. A classification system for clinical relevance of somatic variants identified in molecular profiling of cancer. *Genetics In Medicine* 2015; **18**: 128.

11. Li MM, Datto M, Duncavage EJ et al. Standards and Guidelines for the Interpretation and Reporting of Sequence Variants in Cancer: A Joint Consensus Recommendation of the Association for Molecular Pathology, American Society of Clinical Oncology, and College of American Pathologists. *The Journal of Molecular Diagnostics* 2017; **19**: 4-23.

12. Lunter G, Goodson M. Stampy: a statistical algorithm for sensitive and fast mapping of Illumina sequence reads. *Genome research* 2011; **21**: 936-9.

13. Rimmer A, Phan H, Mathieson I et al. Integrating mapping-, assembly- and haplotype-based approaches for calling variants in clinical sequencing applications. *Nature genetics* 2014; **46**: 912-8.

14. Lek M, Karczewski KJ, Minikel EV et al. Analysis of protein-coding genetic variation in 60,706 humans. *Nature* 2016; **536**: 285-91.

15. Karczewski KJ, Francioli LC, Tiao G et al. Variation across 141,456 human exomes and genomes reveals the spectrum of loss-of-function intolerance across human protein-coding genes. *bioRxiv* 2019: 531210.

16. Popitsch N, Consortium WGS, Schuh A et al. ReliableGenome: annotation of genomic regions with high/low variant calling concordance. *Bioinformatics (Oxford, England)* 2017; **33**: 155-60.

17. Richards S, Aziz N, Bale S et al. Standards and guidelines for the interpretation of sequence variants: a joint consensus recommendation of the American College of Medical Genetics and Genomics and the Association for Molecular Pathology. *Genetics In Medicine* 2015; **17**: 405.

18. Sobreira N, Schiettecatte F, Valle D et al. GeneMatcher: a matching tool for connecting investigators with an interest in the same gene. *Human mutation* 2015; **36**: 928-30.
